# The early stage of COVID-19 pandemic: Gastrointestinal manifestations and liver injury in COVID-19 patients in Wuhan, China

**DOI:** 10.3389/fmed.2022.997000

**Published:** 2022-10-20

**Authors:** Dafan Chen, Min Ning, Yun Feng, Jun Liu

**Affiliations:** ^1^Department of Gastroenterology, Shanghai General Hospital, Shanghai Jiao Tong University School of Medicine, Shanghai, China; ^2^Digestive Endoscopic Center, Shanghai Sixth People's Hospital Affiliated to Shanghai Jiao Tong University School of Medicine, Shanghai, China; ^3^Department of Nephrology, Shanghai General Hospital, Shanghai Jiao Tong University School of Medicine, Shanghai, China

**Keywords:** COVID-19, gastrointestinal symptoms, liver injury, Wuhan, early stage

## Abstract

There are few and inconsistent data focusing on gastrointestinal (GI) manifestations and liver injury in China's early stage of COVID-19 pandemic. In this study, we research the prevalence and role of GI symptoms and liver injury in COVID-19 patients in Wuhan during the disease's first outbreak. We conducted a cross-sectional observational study in a non-ICU unit in Wuhan, China. COVID-19 patients were consecutively admitted from 23 February 2020 to 5 April 2020. Demographic and clinical data were retrieved and analyzed throughout the disease course. A total of 93 patients were enrolled, including 45.2% moderate, 54.8% severe, and 2.2% critical type patients. 69.9% of patients had at least one GI symptom; if excluding hyporexia/anorexia, 49.5% of patients showed at least one GI symptom. The incidence rate of hyporexia/anorexia, diarrhea, nausea/vomiting, abdominal discomfort/pain, and elevated liver enzymes were 67.7, 29.0, 28.0, 21.5, and 23.7%, respectively. Patients with GI symptoms or elevated liver enzymes have a higher risk of severe type disease than patients without GI symptoms or elevated liver enzymes (67.7 vs. 25.0%, *p* < 0.001; 77.3 vs. 47.9%, *p* = 0.016, respectively), and experienced longer disease duration. In multivariate analysis, hyporexia/anorexia was confirmed as an independent predictive factor of severe type disease (odds ratio: 5.912; 95% confidence interval: 2.247–15.559; *p* < 0.001). In conclusion, in the early stage of the COVID-19 pandemic, GI symptoms and elevated liver enzymes are common throughout the disease course, and associated with severer disease and longer disease duration.

## Introduction

The coronavirus-associated disease 2019 (COVID-19) first broke out in Wuhan, China, in December 2019 and has spread worldwide. The pathogen responsible for COVID-19 was identified as severe acute respiratory syndrome coronavirus 2 (SARS-CoV-2) (1). The common symptoms of COVID-19 include fever, cough, shortness of breath and other respiratory symptoms (2). Many studies have shown that some COVID-19 patients also had gastrointestinal (GI) manifestations and liver injury, including hyporexia/anorexia, nausea, vomiting, diarrhea, abdominal discomfort, elevated liver enzymes and so on (3–6).

However, there are inconsistent data of GI manifestations and liver injury in COVID-19, especially in China's early stage of COVID-19 pandemic. Limited studies in the early COVID-19 pandemic about the prevalence of GI manifestations and liver injury varied greatly (2, 7–9). Most of the studies were retrospective, and reported the GI symptoms and liver enzymes only at admission, but not throughout the disease course of COVID-19. Moreover, many studies investigated various clinical characteristics of COVID-19, and clinicians may not pay much attention to the GI manifestations and liver injury. So, the prevalence of GI symptoms and liver injury may be underestimated. The relationship between GI symptoms and disease severity was inconsistent. During the early COVID-19 pandemic, several studies focusing on GI symptoms showed that COVID-19 with GI symptoms had a higher risk of severe illness and intensive care unit (ICU) admission in China (8, 10). However, other studies found that patients with GI symptoms were not severer (4, 11, 12). Studies also showed that COVID-19 could have hepatic involvement, usually characterized by elevated liver enzymes though jaundice was uncommon (5, 13). Although some reports suggested that COVID-19 patients with elevated liver enzymes may have severer disease course, the result in other studies was inconsistent (5, 14, 15).

Investigating the situation of GI manifestations and liver injury in the early stage of the COVID-19 pandemic is helpful to know the change in disease characteristics with time, the impact of virus mutation on the disease development, and the measures for improving the COVID-19 course. In this study, we carried out a cross-sectional observational study, and focused on GI symptoms and liver injury throughout the disease course of COVID-19 in a non-ICU referral unit in Wuhan, China during the period of the disease's first outbreak.

## Methods

### Study design

This was a cross-sectional observational study conducted in a non-ICU referral unit in Leishenshan Hospital, Wuhan, China, which is a designated newly established hospital to treat COVID-19 patients. This non-ICU referral unit was taken over by Shanghai General Hospital. The institutional ethics board of Shanghai General Hospital of Shanghai Jiao Tong University School of Medicine and Leishenshan Hospital approved this study. This ward received confirmed moderate and severe type of COVID-19 patients but not mild and critical cases, who were transferred from other hospitals. Diagnosis of COVID-19 was confirmed by nasopharyngeal or throat swab testing for SARS-CoV-2. The classification of COVID-19 patients and discharge criteria were according to the seventh edition of the COVID-19 diagnosis and treatment guideline of the National Health Commission of China. The classification criteria are as follows: mild type: clinical symptoms were mild and imaging showing no pneumonia; moderate type: have symptoms of fever, respiratory tract inflammation, and imaging showing pneumonia; severe type, meet any of the following criteria: dyspnea and respiratory rate ≥30 beats/min in the resting state, oxygen saturation ≤ 93% in the resting state, arterial blood oxygen partial pressure/fraction of inspired oxygen concentration ≤ 300 mmHg, lung imaging showed that the lesion progressed more than 50% within 24–48 h; Critical type, meet any of following criteria: respiratory failure requiring mechanical ventilation, shock, combined with other organs failure requiring ICU monitoring and treatment. Patients discharged must meet all the following criteria: body temperature returned to normal for more than 3 days, respiratory symptoms improved significantly, pulmonary imaging showed that the acute exudative lesions improved significantly, the SARS-CoV-2 nucleic acid test of respiratory tract samples was negative for two consecutive times (the sampling interval was more than 24 h).

In-patients who were consecutively admitted between 23 February 2020 and 5 April 2020 were included. If patients can't offer specific information regarding the digestive symptoms, or had active GI diseases before COVID-19 onset, such as inflammatory bowel disease, peptic ulcer and gastroesophageal reflux disease, or suffered from active liver disease before COVID-19 onset, the cases were excluded. The enrolled patients were followed up until discharge.

The primary outcome is the prevalence of GI symptoms and elevation of liver enzymes throughout the disease's course of COVID-19. The secondary outcome was to evaluate the correlation of GI symptoms and elevation of liver enzymes with disease classification and the disease duration.

### Data collection

The relevant data was obtained from clinical medical charts and face-to-face interviews. Demographic and clinical data were collected from disease onset to discharge, which included gender; age; comorbidity histories (previous medical records, past drug history); smoking status; alcohol drinking; date of disease onset; respiratory symptoms (cough, dyspnea); digestive symptoms (hyporexia/anorexia, nausea, vomiting, diarrhea, abdominal pain/discomfort); systemic symptoms (fever, fatigue); date of disease diagnosis; disease duration; laboratory test results (complete blood count, C-reactive protein, liver function, renal function, coagulation, etc); chest CT scan; treatment measures. Diarrhea was defined as the passage of three or more stools per day, which meet the types 5–7 according to seven-item Bristol stool scale (16, 17). The disease duration was considered as the time from disease onset to discharge.

### Statistical analysis

Continuous variables were expressed as mean ± standard deviation or median with the range in normally and abnormally distributed data. The differences between two groups were compared by Student's *t*-test for normally distributed data or Wilcoxon rank sum test for abnormally distributed data. Categorical variables were expressed as frequency rates or percentages, and analyzed by chi-square test or Fisher's exact test. In the multivariate analysis, stepwise logistic regression analysis or liner regression analysis was used. A two-tailed *p*-value < 0.05 was considered statistically significant. All statistical analyses were conducted with SPSS software (version 19.0, IBM, Chicago, USA).

## Results

### Demographic and clinical characteristics of patients

A total of 95 consecutive patients were admitted to this ward during the study period. All patients can offer specific information regarding the whole disease course. Two of them who had active gastroesophageal reflux disease or peptic ulcer before COVID-19 onset were excluded. Finally, 93 patients (47 women and 46 men) were included in this study with an average age of 58.0 ± 12.1 years. The enrolled population had 42 (45.2%) moderate type patients and 51 (54.8%) severe type patients. During the study period, two severe type patients with GI symptoms were transferred to ICU for aggravation of illness, and one of two had chronic renal failure, the other one had coronary heart disease. A large proportion of patients had at least one comorbidity (60.2%). The most frequent comorbidity was hypertension (39.8%), diabetes mellitus (15.1%), cardiac disease (8.6%), and cerebrovascular disease (8.6%). All patients were cured and discharged.

In our cohort, two patients presented anorexia as first symptom, one patient only showed anorexia without fever and respiratory symptoms, other patients developed GI symptoms at the same time of or after presenting fever or the respiratory symptoms, and 77 (82.8%) patients had respiratory symptoms including cough and dyspnea. Of enrolled patients, 65 patients (69.9%) showed at least one GI symptom, which presented as follows: 63 (67.7%) hyporexia/anorexia, 27 (29.0%) diarrhea, 26 (28.0%) nausea or vomiting, 20 (21.5%) abdominal discomfort/pain. If hyporexia/anorexia is excluded from GI symptoms because it is less specific for the GI tract, 46 (49.5%) patients showed at least one GI symptom. Twenty-two patients (23.7%) presented with abnormal liver function, including 22 patients with elevated ALT, 14 patients with elevated AST, and four patients with slightly elevated hemobilirubin. All abnormal liver function were found after the onset of COVID-19. Demographic and clinical parameters in patients were shown in Table 1. The details of patients are in the Supplementary material.

**Table 1:** Demographic and clinical parameters in patients with and without GI symptoms or elevated liver enzyme.

**Parameters**	**Enrolled patients (*n* = 93)**	**GI symptoms**		***p*-Value**	**Elevated liver enzyme**		***p*-Value**
		**With (*n* = 65)**	**Without (*n* = 28)**		**With (*n* = 22)**	**Without (*n* = 71)**	
Age, years	58.0± 12.1	58.7 ± 11.8	56.3 ± 13.0	0.397	56.5 ± 12.6	58.4 ± 12.0	0.509
Male	46	30	16	0.331	12	34	0.585
Smoking	9	5	4	0.324	4	5	0.208
Alcohol	5	2	3	0.159	2	3	0.589
**Comorbidities**							
None	37	26	11	0.949	9	28	0.902
Any comorbidities	56	39	17		13	43	
HTN	37	26	11	0.949	9	28	0.902
DM	14	10	4	1.000	1	13	0.175
COPD	6	3	3	0.361	2	4	0.624
Cardiac disease	8	5	3	0.693	2	6	1.000
Cerebrovascular disease	8	5	3	0.693	0	8	0.191
CKD	5	5	0	0.131	1	4	1.000
CLD	6	3	3	0.361	1	5	1.000
Malignancy	1	0	1	0.301	0	1	1.000
**Symptoms**							
Fever	73	52	21	0.590	18	55	0.774
Cough	70	51	19	0.277	18	52	0.415
Shortness of breath	53	45	8	<0.001	17	36	0.028
Fatigue	59	52	7	<0.001	18	41	0.041
**Disease classification**							
Mild type	0	0	0	<0.001	0	0	0.016
Moderate type	42	21	21		5	37	
Severe (including critical) type	51 (2)	44 (2)	7 (0)		17 (1)	34 (1)	
**Treatments**							
Antibiotics treatment	35	25	10	0.802	11	24	0.171
Antiviral treatment	90	63	27	1.000	22	68	1.000
Glucocorticoid treatment	7	6	1	0.670	5	2	0.007
Duration of disease, days	54.3± 13.4	56.4 ± 12.7	49.6 ± 14.1	0.026	59.3 ± 14.9	52.8 ± 12.6	0.046

HTN, hypertension; DM, diabetes mellitus; COPD, chronic obstructive pulmonary disease; CKD, chronic kidney disease; CLD, chronic liver disease.

### The relationship between GI symptoms or elevation of liver enzymes and clinical characteristics

**Figure 1:**
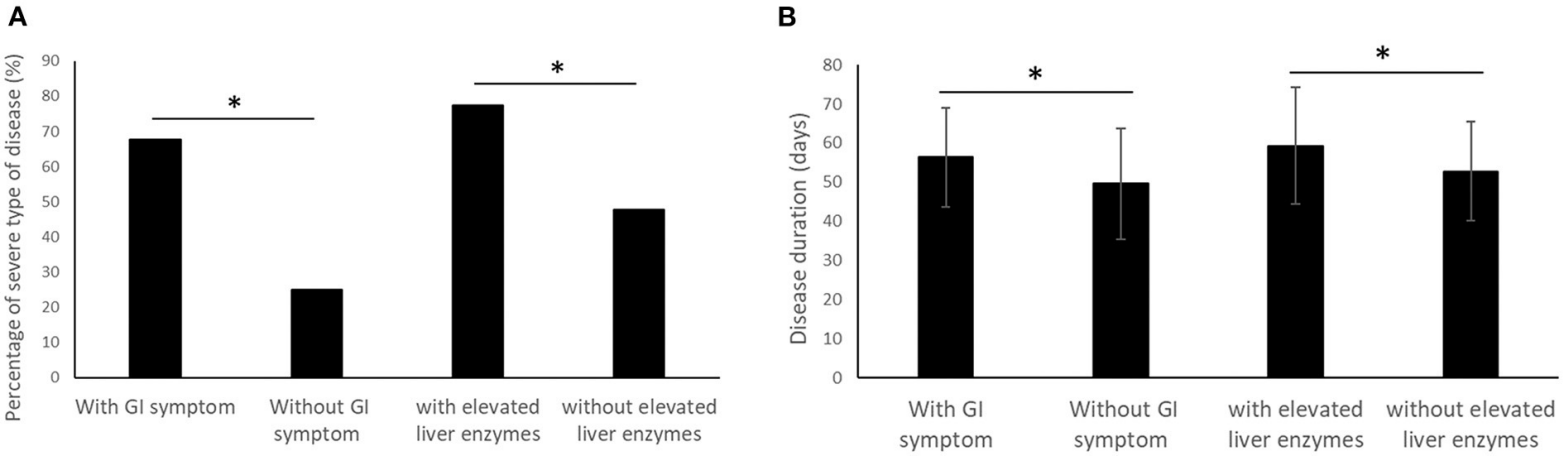
Comparison of percentage of severe type of disease **(A)** and disease duration **(B)** between patients with and without GI symptom/elevated liver enzymes. (*p < 0.05).

Whether or not hyporexia/anorexia was included in GI symptoms, patients with GI symptoms have a significantly higher risk of severe type disease than patients without GI symptoms (67.7 vs. 25.0%, *p* < 0.001, including hyporexia/anorexia, Figure 1A; 71.7 vs. 38.3%, *p* = 0.001, not including hyporexia/anorexia). Compared with patients without GI symptoms, patients with GI symptoms showed a higher rate of shortness of breath (*p* < 0.001) and fatigue (*p* < 0.001), lower blood hemoglobin (*p* = 0.001) and albumin (*p* = 0.003), higher blood d-Dimer (*p* = 0.001) and urea nitrogen (*p* = 0.014), and longer disease duration (*p* = 0.026, Figure 1B). Patients with elevated liver enzymes showed a significantly higher risk of severe type disease (77.3 vs. 47.9%, *p* = 0.016, Figure 1A), fatigue (*p* = 0.041), glucocorticoid treatment (*p* = 0.007), lower albumin (*p* = 0.005), and longer disease duration (*p* = 0.046, Figure 1B). The results of the comparison in demographic, clinical and laboratory test parameters were shown in Tables 1, 2. In the subgroup analysis, hyporexia/anorexia, diarrhea and abdominal discomfort/pain were all associated with significantly higher risk of severe type disease (*p* < 0.00, *p* = 0.017, and *p* = 0.011, respectively). Patients with hyporexia/anorexia, diarrhea or nausea/vomiting have a higher risk of fatigue, and lower blood hemoglobin. Specific subgroup analysis data are shown in Table 3.

**Table 2:** Laboratory test in patients with and without GI symptoms or elevated liver enzyme.

**Parameters**	**Enrolled patients (*n* = 93)**	**GI symptoms**		***p*-Value**	**Elevated liver enzyme**		***p*-Value**
		**With (*n* = 65)**	**Without (*n* = 28)**		**With (*n* = 22)**	**Without (*n* = 71)**	
WBC, × 10^9^ cells/L	6.1 (5.0, 7.4)	6.0 (5.0, 7.4)	6.1 (5.3, 7.5)	0.471	6.8 (5.2, 8.2)	5.9 (5.0, 7.0)	0.071
LYM, × 10^9^ cells/L	1.4 (1.1, 1.8)	1.4 (1.0, 1.7)	1.5 (1.2, 1.8)	0.188	1.4 (1.0, 1.8)	1.4 (1.2, 1.8)	0.735
Neutrophil, × 10^9^ cells/L	3.6 (2.7, 4.6)	3.5 (2.7, 4.5)	3.7 (2.8, 4.6)	0.642	4.2 (3.4, 5.6)	3.4 (2.6, 4.5)	0.046
HB, g/L	118.0 (109.5, 128.0)	116.0 (107.5, 124.5)	126.5 (116.8, 137.5)	0.001	118.0 (107.3, 126.8)	118.0 (110.0, 128.0)	0.769
PLT, × 10^9^/L	251.0 (202.5, 306.0)	252.0 (203.0, 336.5)	228.0 (192.5, 286.5)	0.258	278.0 ± 112.4	253.5 ± 85.7	0.281
CRP, mg/L	1.9 (0.7, 7.1)	1.9 (0.7, 7.3)	2.1 (0.7, 4.7)	0.615	2.0 (0.7, 7.3)	1.9 (0.6, 6.6)	0.618
PT, s	11.3 (11.0, 11.8)	11.3 (10.9, 11.8)	11.3 (11.0, 11.9)	0.967	11.3 (11.0, 11.8)	11.3 (11.0, 11.8)	0.986
APTT, s	26.8 ± 4.2	26.7 ± 4.3	27.0 ± 3.9	0.795	26.9 ± 4.3	26.7 ± 4.2	0.853
FI, g/L	2.8 (2.4, 3.3)	2.9 (2.5, 3.3)	2.7 (2.3, 3.6)	0.763	2.6 (2.3, 3.0)	2.9 (2.5, 3.7)	0.191
D-dimer, mg/L	0.5 (0.3, 1.1)	0.7 (0.3, 1.2)	0.3 (0.2, 0.6)	0.001	0.8 (0.4, 1.1)	0.5 (0.2, 1.0)	0.123
ALT, U/L	30.0 (18.0, 47.0)	30.0 (18.5, 63.0)	29.5 (17.0, 36.5)	0.243	65.5 (43.0, 150.5)	24.0 (16.0, 35.0)	<0.001
AST, U/L	21.0 (16.5, 29.0)	23.0 (16.5, 33.0)	19.5 (16.3, 23.0)	0.119	38.5 (23.5, 67.5)	20.0 (16.0, 24.0)	<0.001
TBIL, μmol/L	10.2 (8.0, 14.8)	10.3 (7.8, 15.0)	10.2 (8.6, 13.0)	1.000	11.5 (7.3, 15.6)	10.0 (8.0, 13.2)	0.684
ALB, g/L	37.9 (34.9, 39.5)	37.7 (34.1, 38.5)	38.8 (37.0, 41.2)	0.003	36.3 (33.0, 38.0)	38.1 (35.7, 40.1)	0.005
BUN, mmol/L	5.2 (4.3, 6.4)	5.5 (4.3, 6.7)	4.9 (4.2, 5.3)	0.014	5.5 (4.9, 6.6)	5.0 (4.2, 6.4)	0.204
CR, μmol/L	65.0 (55.4, 65.7)	66.8 (54.7, 79.6)	61.7 (55.5, 73.7)	0.276	64.0 (53.6, 77.2)	65.0 (55.2, 75.8)	0.728
UA, μmol/L	324.3 ± 94.9	320.2 ± 95.0	333.9 ± 95.5	0.526	337.0 ± 111.6	320.4 ± 89.6	0.476

WBC, white blood cell; LYM, Lymphocyte; HB, hemoglobin; PLT, platelet; CRP, C-reactive protein; PT, prothrombin time; APTT, activated partial thromboplastin time; FI, fibrinogen; ALT, alanine aminotransferase; AST, aspartate aminotransferase; TBIL, total bilirubin; ALB, albumin; BUN, blood urea nitrogen; CR, creatinine; UA, uric acid.

**Table 3:** The subgroup analysis of patients with and without GI symptoms.

**Parameters**	**Hyporexia** **/anorexia**		***p*-Value**	**Nausea** **/vomiting**		***p*-Value**	**Diarrhea**		***p*-Value**
	**With**	**Without**		**With**	**Without**		**With**	**Without**	
Age, years	59.0 ± 11.8	55.7 ± 12.7	0.225	55.6 ± 11.2	58.9 ± 12.4	0.249	57.6 ± 11.2	58.1 ± 12.6	0.854
Male	30	16	0.606	12	34	0.691	10	36	0.125
**Symptoms**									
Fever	50	23	0.767	18	55	0.176	22	51	0.654
Cough	49	21	0.416	20	50	0.818	22	48	0.374
Shortness of breath	44	9	<0.001	18	35	0.137	20	33	0.033
Fatigue	50	9	<0.001	21	38	0.031	23	36	0.005
Disease classification			<0.001			0.082			0.017
Mild type	0	0		0	0		0	0	
Moderate type	20	22		8	34		7	35	
Severe (including critical) type	43 (2)	8		18 (2)	33		20 (2)	31	
**Laboratory test**									
WBC count, × 10^9^ cells per L	6.0 (5.0, 7.3)	6.1 (5.3, 7.6)	0.318	6.0 (4.9, 7.8)	6.1 (5.1, 7.4)	0.915	5.2 (4.9, 6.7)	6.1 (5.2, 7.6)	0.061
LYM count, × 10^9^ cells per L	1.4 (1.0, 1.7)	1.5 (1.2, 1.8)	0.141	1.5 (1.0, 1.7)	1.4 (1.1, 1.8)	0.726	1.6 (1.1, 1.8)	1.4 (1.1, 1.8)	0.488
Neutrophil count, × 10^9^ cells per L	3.4 (2.7, 4.5)	3.9 (3.0, 4.7)	0.430	3.8 (2.6, 4.7)	3.5 (2.8, 4.6)	0.811	3.1 (2.6, 4.3)	3.8 (3.0, 4.8)	0.039
HB, g/L	116.0 (108.0, 125.0)	123.5 (112.8, 136.5)	0.004	108.5 ± 21.9	121.1 ± 12.8	0.009	111.8 ± 18.4	120.0 ± 15.5	0.031
PLT, × 10^9^/L	253.0 (203.0, 339.0)	228.0 (187.8, 283.5)	0.155	297.3 ± 104.4	244.5 ± 83.9	0.013	234.0 (185.0, 334.0)	254.5 (202.8, 305.5)	0.617
PT, s	11.3 (10.9, 11.8)	11.3 (11.0, 11.8)	0.961	11.4 (11.0, 12.0)	11.3 (10.9, 11.7)	0.370	11.2 (10.8, 11.9)	11.4 (11.0, 11.7)	0.358
APTT, s	26.8 ± 4.4	26.8 ± 3.8	0.930	28.1 ± 4.4	26.3 ± 4.0	0.068	25.9 ± 4.2	27.2 ± 4.1	0.180
FI, g/L	2.8 (2.4, 3.4)	2.8 (2.3, 3.4)	0.850	2.8 (2.5, 3.4)	2.9 (2.4, 3.3)	0.969	2.8 (2.5, 3.0)	2.8 (2.4, 3.8)	0.446
d-dimer, mg/L	0.8 (0.3, 1.3)	0.3 (0.2, 0.6)	<0.001	0.7 (0.3, 1.2)	0.4 (0.3, 1.0)	0.204	0.7 (0.3, 1.2)	0.4 (0.2, 1.0)	0.253
ALT, U/L	30.9 (19.0, 64.0)	27.0 (16.3, 35.5)	0.136	33.0 (19.0, 91.0)	28.0 (17.0, 39.0)	0.101	25.0 (17.0, 64.0)	31.0 (18.0, 46.3)	0.783
AST, U/L	23.0 (17.0, 35.0)	19.5 (16.0, 23.0)	0.126	24.5 (17.8, 55.3)	20.0 (16.0, 27.0)	0.081	22.0 (16.0, 35.0)	21.0 (16.8, 27.5)	0.599
TBIL, μmol/L	10.3 (7.9, 14.8)	10.2 (8.1, 13.8)	0.987	10.1 (7.5, 16.7)	10.2 (8.4, 13.4)	0.881	9.6 (7.2, 15.2)	10.3 (8.8, 13.6)	0.729
ALB, g/L	37.7 (33.9, 38.6)	38.5 (36.7, 40.9)	0.004	37.8 (35.5, 38.6)	37.9 (34.8, 40.2)	0.394	38.0 (34.9, 38.6)	37.9 (34.7, 39.8)	0.856
Duration of disease, days	56.7± 12.7	49.5 ± 13.8	0.015	58.1 ± 12.3	52.9 ± 13.6	0.094	59.8 ± 13.4	52.1 ± 12.9	0.012

WBC, white blood cell; LYM, lymphocyte; HB, Hemoglobin; PLT, platelet; PT, prothrombin time; APTT, activated partial thromboplastin time; FI, fibrinogen; ALT, alanine aminotransferase; AST, aspartate aminotransferase; TBIL, total bilirubin; ALB, albumin.

At multivariate analysis (including age, gender, comorbidities, hyporexia/anorexia, diarrhea, nausea/vomiting, abdominal discomfort/pain and elevation of liver enzymes), hyporexia/anorexia was confirmed as an independent predictive factor of severe type disease in the logistic regression analysis [odds ratio (OR): 5.912; 95% confidence interval (CI): 2.247–15.559; *p* < 0.001], and diarrhea was confirmed as an independent predictive factor of longer disease duration in the liner regression analysis (*p* = 0.012). In addition, to study the relationship between GI symptoms or liver injury and other factors other than COVID-19, we included some factors (including age, gender, fever, comorbidities) for multivariate analysis. We found no significant correlation between these factors and GI symptoms or liver injury.

## Discussion

Patients with COVID-19 often present with respiratory symptoms, and can have GI symptoms and liver injury (14). However, there are few and inconsistent data on GI manifestations and liver injury in the early stage of the COVID-19 pandemic. Therefore, understanding the prevalence of GI symptoms and liver dysfunction and the relationship between GI symptoms or liver dysfunction and COVID-19 course in the early COVID-19 pandemic is helpful to investigate the variation of disease characteristics with virus mutation and measures for treating COVID-19.

This is an observational cross-sectional study conducted in a non-ICU referral unit in Wuhan during the course of the COVID-19 first outbreak. We investigated the GI symptoms and liver injury throughout the COVID-19 course. We found that 69.9% patients showed at least one GI symptom, and 23.7% patients experienced elevation of liver enzymes. The most common GI symptoms were hyporexia/anorexia, followed by diarrhea, nausea/vomiting and abdominal discomfort/pain. Even if hyporexia/anorexia was excluded from GI symptoms for less specific to the GI tract, about half of the patients presented at least one GI symptom. The manifestations of GI symptoms are similar to published reports. However, the prevalence of GI symptoms and liver enzyme elevation in our study is higher than some published studies in the early COVID-19 pandemic (2, 18, 19). The difference between our results and published reports may be partly related to the different attention to GI symptoms among the clinicians. Moreover, we investigate GI symptoms and liver enzyme in the whole course of disease, but most published studies focus on the GI symptoms and liver enzyme only at admission which could cause underestimating the prevalence. So, this study can offer results closer to the actual situation. As the pandemic spread, comparative analysis by patient location showed a significantly higher proportion of GI symptom and abnormal liver enzymes in the non-China subgroup (6). At present, there is a lack of detailed information of GI symptom and liver injury at patients infected with different virus variants, such as Omicron or Delta variants. Further investigation is needed to study if the virus mutation as spread might affect its tropism for the digestive tract and liver.

Published studies about the relationship between GI symptoms or elevation of liver enzymes and clinical characteristics came to inconsistent results in the early COVID-19 pandemic. Several reports showed that COVID-19 patients with GI symptoms or elevation of liver enzymes were severer with higher ICU admission (8, 10, 14, 15). Conversely, some studies considered that COVID-19 patients with GI symptoms have less lung involvement and better prognosis (4, 11, 12). In our study, we found that COVID-19 patients with GI symptoms or elevation of liver enzymes experienced severer clinical course, with a higher rate of severe type of disease, lower blood hemoglobin and albumin, and longer disease duration. Furthermore, hyporexia/anorexia was an independent predictive factor of severe type disease, and diarrhea was an independent predictive factor of longer disease duration. GI symptoms can affect the intake and absorption of nutrients, which is related to low hemoglobin and albumin. Liver dysfunction can complicate the disease. These factors can aggravate the COVID-19. The cause of GI symptoms or elevation of liver enzymes is not clear at present. Hyporexia/anorexia may be part of the systemic inflammatory response, which is involved in the action of cytokines and microbial products on the central nervous system (20). SARS-CoV-2 could infect cells through the angiotensin-converting enzyme 2 receptor, expressed in the GI tract and liver cells (21, 22). The mechanisms of GI symptoms and liver injury may include direct infection of SARS-CoV-2, side effects of drugs, gut microbiota disorder and inflammatory immune response (23–25). Therefore, understanding of these mechanisms could help us to better manage those patients with GI symptoms and liver injury.

There are some limitations in our study. Firstly, our cohort mainly included only moderate and severe types of patients in a non-ICU ward who were relatively old and often had comorbidities, and can't represent all COVID-19 patients. However, even if mild type of patients is included, the occurrence of GI symptoms is also common in some reports (26). Secondly, this study did not differentiate the GI symptoms and liver injury at different stages of the disease. Finally, this study enrolled a relatively small sample size, and carried out at a hospital which only received patients transferred from other hospitals. So, a selection bias in the COVID-19 population is inevitable.

Our study design also presents strengths. We focus on GI symptoms and liver injury in a group of patients in Wuhan during the course of the disease's first outbreak, which lacked in-depth investigation. And we carried out a cross-sectional observational study, which can increase information accuracy compared with retrospective study. Moreover, we have investigated the incidence of GI symptoms and liver enzyme elevation throughout the course of the COVID-19. In contrast, many published studies have only reported the occurrence at or before admission.

In conclusion, this cross-sectional observational study presented results closer to the actual situation in the early stage of COVID-19 pandemic. It showed that GI symptoms and elevated liver enzymes are common throughout the disease course, and associated with severer disease and longer disease duration.

## Data availability statement

The datasets presented in this study can be found in online repositories. The names of the repository/repositories and accession number(s) can be found in the article/Supplementary material.

## Ethics statement

The studies involving human participants were reviewed and approved by the Institutional Ethics Board of Shanghai General Hospital of Shanghai Jiao Tong University School of Medicine and Leishenshan Hospital. Written informed consent for participation was not required for this study in accordance with the national legislation and the institutional requirements.

## Author contributions

DC and JL: design of the study and editing the article. DC, YF, and MN: acquisition of data and statistical analysis. DC and MN: drafting the article. All authors contributed to the article and approved the submitted version.

## Conflict of interest

The authors declare that the research was conducted in the absence of any commercial or financial relationships that could be construed as a potential conflict of interest.

## Publisher's note

All claims expressed in this article are solely those of the authors and do not necessarily represent those of their affiliated organizations, or those of the publisher, the editors and the reviewers. Any product that may be evaluated in this article, or claim that may be made by its manufacturer, is not guaranteed or endorsed by the publisher.
